# Association of Allostatic Load With Overall Mortality Among Patients With Metastatic Non–Small Cell Lung Cancer

**DOI:** 10.1001/jamanetworkopen.2022.21626

**Published:** 2022-07-07

**Authors:** Samilia Obeng-Gyasi, Yaming Li, William E. Carson, Sarah Reisinger, Carolyn J. Presley, Peter G. Shields, David P. Carbone, DuyKhanh P. Ceppa, Ruth C. Carlos, Barbara L. Andersen

**Affiliations:** 1 Division of Surgical Oncology, Department of Surgery, The Ohio State University, Columbus; 2 Department of Biomedical Informatics, University of Pittsburgh, Pittsburgh, Pennsylvania; 3 Division of Medical Oncology, Department of Internal Medicine, The Ohio State University, Columbus; 4 Department of Surgery, Indiana University School of Medicine, Indianapolis; 5 University of Michigan Comprehensive Cancer Center, Ann Arbor; 6 Department of Psychology, The Ohio State University, Columbus

## Abstract

**Importance:**

Adverse social determinants of health (SDHs) (eg, poverty) are associated with poor oncologic outcomes among patients with lung cancer. However, no studies have evaluated biological correlates of adverse SDHs, operationalized as allostatic load (AL), with mortality due to lung cancer.

**Objective:**

To examine the association among AL, SDHs, and mortality among patients with metastatic non–small cell lung cancer (NSCLC).

**Design, Setting, and Participants:**

This cross-sectional study of an observational cohort was performed at a National Cancer Institute–designated comprehensive cancer center with data accrued from June 1, 2017, to August 31, 2019. Patients with metastatic (stage IV) NSCLC enrolled at diagnosis into a prospective observational cohort study were included in the present analysis if they had all the biomarkers to calculate an AL score (N = 143). Follow-up was completed on August 31, 2021, and data were analyzed from July 1 to September 30, 2021.

**Exposures:**

Social determinants of health.

**Main Outcomes and Measures:**

Overall mortality and AL.

**Results:**

A total of 143 patients met the study criteria with a median age of 63 (IQR, 55-71) years (89 men [62.2%] and 54 women [37.8%]). In terms of race and ethnicity, 1 patient (0.7%) was Asian, 7 (4.9%) were Black, 117 (81.8%) were White, 17 (11.9%) were of multiple races, and 1 (0.7%) was of other race or ethnicity. The mean (SD) AL was 2.90 (1.37). Elevated AL covaried with lower educational level (*r* = −0.26; *P* = .002), male sex (*r* = 0.19; *P* = .02), limited mobility (*r* = 0.19; *P* = .04), worsening self-care (*r* = 0.30; *P* < .001), problems engaging in usual activities (*r* = 0.21; *P* = .01), depressive symptoms (*r* = 0.23; *P* = .005), and a high number of stressful life events (*r* = 0.30; *P* < .001). Multivariable analysis found only increasing difficulty with mobility (*r* = 0.37 [95% CI, 0.13-0.60]; *P* = .002) and male sex (*r* = 0.63 [95% CI, 0.19-1.08]; *P* = .005) associated with higher AL. On adjusted analysis, elevated AL (hazard ratio, 1.43 [95% CI, 1.16-1.79]; *P* = .001) and low educational level (hazard ratio, 2.11 [95% CI, 1.03-4.34]; *P* = .04) were associated with worse overall mortality.

**Conclusions and Relevance:**

The findings of this cross-sectional study suggest that higher AL was associated with adverse SDHs and worse overall mortality among patients with advanced NSCLC. These results provide a framework for replication and further studies of AL as a biological correlate for SDH and future prognostic marker.

## Key Points

**Question:**

Is there an association among allostatic load (AL), social determinants of health (SDHs), and overall mortality in patients with metastatic non–small cell lung cancer (NSCLC)?

**Findings:**

In this cross-sectional study of 143 patients with metastatic NSCLC, elevated AL was associated with adverse SDHs and worse overall mortality.

**Meaning:**

These findings suggest that AL should be considered as a biological correlate of SDHs and a prognostic marker for overall mortality among patients with metastatic NSCLC.

## Teaser

This cross-sectional study examines the association of allostatic load, social determinants of health, and overall mortality among patients with metastatic non–small cell lung cancer.

## Introduction

Lung cancer is the leading cause of cancer-related mortality in the US and the second most common cancer among both men and women.^1,2^ Approximately 55% of patients presenting with non−small cell lung cancer (NSCLC), the most common type of lung cancer, will have distant metastasis at the time of diagnosis.^3^ Drivers of lung cancer mortality are a combination of clinical characteristics^4^ and social determinants of health (SDHs).^5^ Social determinants of health are multilevel factors such as socioeconomic and political policy contexts, socioeconomic position, psychosocial factors, behaviors, biological factors, and living and working conditions that predispose to disease and drive inequities in clinical outcomes.^6^ For patients with lung cancer, adverse SDHs such as delay in surgical care,^7^ being unmarried,^8^ lower socioeconomic status,^9^ and continuing depressive symptoms^10^ have been associated with higher mortality. Nonetheless, biological correlates for adverse SDHs and their implications for mortality are unclear and less studied.^11^

Allostatic load (AL) is a concept originated by McEwen and Stellar^12^ in 1993 to explain stress as a contributor to disease initiation and progression. Allostatic load is proposed as the physiologic “wear and tear” secondary to an individual’s exposure to external stressors accumulated throughout the life course.^13^ Allostatic load is operationalized using a composite score derived from biomarkers (eg, blood pressure, albumin level, C-reactive protein level) from multiple physiologic systems (eg, metabolic, immune, cardiac, hematologic). The construct validity for AL is well established,^14,15^ with studies suggesting an association between high AL scores and worsened psychiatric symptoms,^16^ peripheral vascular disease,^17^ cognitive deterioration, and physical decline.^18^ In patients with cancer, high AL scores have been associated with poor tumor characteristics (eg, large tumor size),^19^ higher all-cause mortality, and worse cancer-specific mortality^20^ compared with lower AL scores.

There is also evidence that AL correlates with SDHs. Specifically, adverse SDHs such as workplace stress, low income, poor educational attainment, childhood trauma, and less social support are associated with high AL.^21,22^ Moreover, studies suggest an association among patients facing adverse structural SDHs, historically and intentionally excluded populations in the US (eg, Black women), and high AL.^23^ Cumulatively, these studies on AL, SDHs, and disease suggest AL could serve as a plausible biological correlate for external stressors rooted in adverse SDHs and a prognostic marker for oncologic outcomes such as mortality.^24^

To our knowledge, this study provides the first examination of AL in the context of lung cancer, SDH correlates, and overall mortality. Patients with stage IV NSCLC, the most common lung cancer, were enrolled at diagnosis into a prospective observational cohort study.^25^ Patients completed measures of SDHs, stress, psychological symptoms, and functional status, among others. Medical record data from the diagnostic period provided AL indicators—9 biomarkers—and a composite AL score was calculated as described previously.^26,27,28^ For the analyses, the association between AL and the noted relevant variables was considered; next, the association between AL and overall mortality was examined. We anticipated that AL would covary with low educational attainment and multiple comorbidities, and we hypothesized that higher AL values at the time of diagnosis would be associated with elevated mortality risk.

## Methods

### Study Sample

The Ohio State University Office of Responsible Research Practices approved the procedures for this cross-sectional study. The analysis included patients who provided written consent to enroll in the Beating Lung Cancer in Ohio (BLCIO) protocol.^25^ The study followed the Strengthening the Reporting of Observational Studies in Epidemiology (STROBE) reporting guideline.

The BLCIO prospective cohort study enrolled patients with stage IV NSCLC presenting for treatment at The Ohio State University Comprehensive Cancer Center between June 1, 2017, and August 31, 2019. Inclusion criteria consisted of newly diagnosed pathology-confirmed stage IV NSCLC, any comorbidity, 18 years or older, English-speaking, and willing to provide access to medical records, provide biospecimens, and respond to patient-reported outcome measures. Exclusion criteria consisted of prior treatment with definitive chemoradiotherapy for lung cancer, diagnosis earlier than 90 days at enrollment, receipt of lung cancer treatment for more than 1 month, and presence of disabling hearing, vision, or psychiatric conditions (eg, schizophrenia) preventing consent or completion of patient-reported outcomes in English. Unlike other NSCLC trials, patients were not excluded from the study secondary to high performance status, low functional status, or old age. Within 2 weeks of enrollment, patients were contacted by telephone by trained interviewers who conducted the patient-reported outcome assessment. Each patient received $15 for participation.

Owing to the study focus on AL, patients with any missing AL biomarkers were excluded (n = 40). The final cohort consisted of only patients with values in each of the 9 AL biomarkers (n = 143). Follow-up was completed on August 31, 2021.

### Study Measures

#### Allostatic Load

A composite AL score was calculated as described previously,^26,27,28^ using 9 biomarkers reflective of 4 physiologic systems collected at study entry. The physiologic systems were as follows: (1) metabolic, including body mass index (calculated as weight in kilograms divided by height in meters squared) and creatinine, albumin, and glucose levels; (2) immune, consisting of white blood cell count; (3) cardiac, including systolic blood pressure, diastolic blood pressure, and heart rate; and (4) hematologic, consisting of serum hemoglobin level. The hematologic system was included owing to prior studies suggesting an association between anemia and food insecurity,^29^ the latter of which is an SDH. Moreover, anemia is associated with poor outcomes among patients with lung cancer.^30^ Cutoffs were established using those of prior studies evaluating AL and/or established laboratory thresholds for abnormal values. Variable cutoffs were as follows: (1) body mass index greater than 25.0 or less than 18.0^26^; (2) creatinine level greater than 1.2 mg/dL among women and greater than 1.4 mg/dL among men (to convert to micromoles per liter, multiply by 88.4)^31^; (3) albumin level less than 3.4 g/dL (to convert to grams per liter, multiply by 10)^32^; (4) blood glucose level greater than 110 mg/dL (to convert to millimoles per liter, multiply by 0.0555)^33^; (5) white blood cell count greater than 1100/mL (to convert to ×10^9^ per liter, multiply by 0.001)^34^; (6) systolic blood pressure at least 140 mm Hg^19^; (7) diastolic blood pressure at least 90 mm Hg^19^; (8) heart rate greater than 100 beats/min^35^; and (9) hemoglobin level less than 13.2 g/dL in men and less than 11.6 g/dL in women (to convert to grams per liter, multiply by 10).^36^ A point was assigned if the biomarker value was above normal cutoffs for all biomarkers except albumin and hemoglobin levels and for body mass index less than 18.0, for which a point was assigned if the values were below the normal threshold. All the assigned points were summed, with a possible range from 0 to 9. A higher AL score indicated worse physiologic dysregulation.

#### SDHs and Treatment Variables

##### Sociodemographic Factors and Comorbidities

Sociodemographic information included age, race and ethnicity (collected to enable examinations of intergroup similarities and differences in clinical outcomes), income, educational level (less than high school, high school, or beyond high school), marital status (married or not married), and sex (men or women). Self-identified racial groups included Asian, Black, White, other, and multiple (≥2) categories. The other racial category included individuals who did not identify as Asian, Black, White, or multiple races, who were collapsed into a single category owing to very small numbers. Ethnicity was dichotomized as Hispanic or non-Hispanic. Comorbidity, exclusive of metastatic lung cancer, was quantified using the Charlson Comorbidity Index.^37^

##### Psychological and Functional Status Factors

The Patient Health Questionnaire–9,^38^ Generalized Anxiety Disorder 7,^39^ the Impact of Events Scale–Revised,^40,41^ and Life Events Scale^42^ were used to investigate the association between AL and psychological responses and stress. Psychological responses evaluated included depression and anxiety.

The Patient Health Questionnaire–9 evaluates the frequency of symptoms of major depressive disorder. Nine items were rated on a 0- to 3-point Likert scale and summed, with scores ranging from 0 to 27 and a higher score indicating greater severity. Cutoff values are none or mild (1-7), moderate (8-14), moderate to severe (15-19), and severe (20-27).^38^

The Generalized Anxiety Disorder 7 evaluates symptoms consistent with generalized anxiety disorder.^39^ Seven items were rated on a 0- to 3-point Likert scale and summed, with scores ranging from 0 to 21 and a higher score indicating greater severity. Cutoff values were none (0-4), mild (5-9), moderate (10-14), and moderate to severe (15-21).

Two measures of stress were used. The Impact of Events Scale–Revised^40,41^ is widely used to assess subjective stress caused by traumatic events and has been adapted to measure cancer-specific stress as indicated by intrusive thoughts and avoidant thoughts and behaviors within the past week. Sixteen items are rated on a 0- to 4-point scale. Summed scores range from 0 to 64, with higher scores indicating greater stress. The Life Events Scale was used to evaluate the number of stressful life events within the past year. Stressful events evaluated included the death of a close friend or relative, a major financial difficulty, a divorce or break-up, conflict with children or grandchildren, muggings, robberies, accidents, or similar events. A higher score reflects more stressful events during the past year.^42^ Functional status was assessed using the 3 EuroQol 5-Dimension 5-Level (EQ-5D-5L) items of mobility, self-care, and engagement in usual activities. Each item is rated on a 5-point Likert scale, with 0 indicating no problems; 1, slight problems; 2, moderate problems; 3, severe problems; and 4, unable to do.^38^ Items were summed for a total score with a possible range from 0 to 12, with higher scores indicating worse functional status.

### Statistical Analysis

Data were analyzed from July 1 to September 20, 2021. Descriptive characteristics of the study sample are tabulated as frequencies and continuous variables as means with their SDs or medians with an IQR as appropriate. To determine the association between AL and other relevant independent variables, we conducted 2 analyses. First, correlations between AL and other independent variables were examined. Pearson correlations were used for binary variables, Spearman correlations for ordinal variables, and the η coefficient for variables with at least 3 subcategories. Second, variables significantly correlated (2-sided *P* ≤ .05) with AL were included in regression analyses. Using the identified variables, a stepwise forward regression was conducted to determine a parsimonious model that also provided a statistically significant improvement in fit.

To examine the association between AL and overall mortality, univariable Cox proportional hazards models were used to evaluate each of the study variables (independent variables), including AL (independent variable) and mortality (dependent variable) (eTable 3 in the Supplement). Variables significant (2-sided *P* < .05) in the univariable hazards model were then used in a final, multivariable Cox proportional hazards model. Overall mortality was defined as days from trial enrollment until the date of death due to any cause.

For the sensitivity analysis, the primary analysis was repeated using a composite AL score derived from quartiles. In this approach, the distribution of AL in the study sample was assessed and patients received a point if they were in the worst quartile.^18,23,26^ For example, patients in the 75th percentile for blood pressure received a point. Conversely, those in the lowest quartile (ie, 25th percentile for albumin level), received a point. Statistical analyses were performed using Stata, version 16.0 (StataCorp LLC).

## Results

### Overview of Study Population

Of the 186 patients enrolled in the cohort, 143 met the criteria for this study (89 men [62.2%] and 54 women [37.8%]; median age, 63 [IQR, 55-71] years). In terms of race and ethnicity, 1 patient (0.7%) was Asian, 7 (4.9%) were Black, 117 (81.8%) were White, 17 (11.9%) were of multiple races, and 1 (0.7%) was of other race or ethnicity. Most of the study patients had a high school diploma or less (73 [51.0%]), were married (86 [60.1%]), and had multiple comorbidities (mean [SD] Charlson Comorbidity Index 3.73 [2.24]). The study sample reported mild symptoms of depression (mean [SD] Patient Health Questionnaire–9 score, 6.64 [5.45]) and anxiety (mean [SD] Generalized Anxiety Disorder 7 score, 5.52 [5.58]) (eTable 1 in the Supplement). The mean (SD) AL was 2.90 (1.37). Patient characteristics are provided in Table 1.

**Table 1.** Patient Characteristics

Characteristic	Patient data (N = 143)^a^
Age, median (IQR), y	63 (55-71)
Sex	
Women	54 (37.8)
Men	89 (62.2)
Race	
Asian	1 (0.7)
Black	7 (4.9)
White	117 (81.8)
Multiple races	17 (11.9)
Other^b^	1 (0.7)
Hispanic ethnicity	
No	140 (97.9)
Yes	3 (2.1)
Income	
≤$35 000 (low)	49 (34.3)
$35 001-$100 000 (middle)	55 (38.5)
≥$100 001 (high)	24 (16.8)
Missing	15 (10.5)
Education	
Less than high school	19 (13.3)
High school	54 (37.8)
Beyond high school	70 (49.0)
Marital status	
Not married	57 (39.9)
Married	86 (60.1)
Charlson Comorbidity Index, mean (SD)	3.73 (2.24)
Smoker status	
Never	12 (8.4)
Prior	104 (72.7)
Current	27 (18.9)
Alcohol use	
No	73 (51.0)
Yes	70 (49.0)
Subsequent treatment	
Chemotherapy	20 (14.0)
Chemotherapy and immunotherapy	44 (30.8)
Immunotherapy	32 (22.4)
Targeted	30 (21.0)
Missing	17 (11.9)
Follow-up, mean (SE), mo	23.2 (1.5)
Overall survival, mean (SE), mo	15.9 (2.5)

^a^ Unless indicated otherwise, data are expressed as No. (%) of patients. Percentages have been rounded and may not total 100.

^b^ Consists of individuals who did not identify as Asian, Black, White, or multiple races.

### Correlation Between Study Variables and AL

Importantly, the AL scores approximated a normal distribution (eFigure 1 in the Supplement). The AL scores were significantly correlated with SDHs (Table 2), including income, educational level, and sex. In addition, AL was correlated with depressive symptoms, stress (cancer-specific and life stress), and functional status. Specifically, educational achievement (*r* = −0.23; *P* = .006), income (*r* = −0.20; *P* = .02), smoker status (*r* = 0.17; *P* = .04), and treatment received (*r* = −0.33; *P* = .002) inversely correlated with AL. Male sex was correlated with AL (*r* = 0.19; *P* = .02). Higher Patient Health Questionnaire–9 scores (*r* = 0.23; *P* = .005) and a higher number of stressful life events (*r* = 0.30; *P* < .001) were correlated with increasing AL. Increasing difficulty with mobility (*r* = 0.187; *P* = .04), worsening self-care (*r* = 0.30; *P* < .001), and problems engaging in usual activities (*r* = 0.21; *P* = .01), as measured with EQ-5D-5L items, were correlated with higher AL scores. Notably, the Charlson Comorbidity Index and AL were correlated but did not reach statistical significance (*r* = 0.06; *P* = .46). However, with AL as quartiles, there was a correlation between AL and the Charlson Comorbidity Index on univariable analysis (*r* = 0.23; *P* < .001) (eTable 2 in the Supplement) and multivariable analysis (*r* = 0.21 [95% CI, 0.11-0.31]; *P* < .001). On multivariable analysis, stepwise forward regression, only increasing difficulty with mobility (*r* = 0.37 [95% CI, 0.13-0.60]; *P* = .002) and male sex (*r* = 0.63 [95% CI, 0.19-1.08]; *P* = .005) remained associated with increased AL scores (Table 3).

**Table 2.** Correlation Between AL and Descriptive Characteristics, Psychological Variables, and Health Measures

Variable	Correlation coefficient, *r*	*P* value
Age	−0.04	.65
Sex	0.19	.02
Race	0.23	.11
Hispanic ethnicity	0.01	.89
BMI	0.05	.58
Income	−0.20	.02
Educational level	−0.23	.006
Marital status	0.04	.62
Charlson Comorbidity Index	0.06	.46
Smoker status	0.17	.04
Alcohol use	−0.1099	.24
Treatment	−0.333	.002
Patient Health Questionnaire–9	0.235	.005
Generalized Anxiety Disorder 7	−0.035	.68
Impacts of Events Scale	0.25	.003
Life Events Scale	0.30	<.001
EQ-5D-5L		
Mobility	0.19	.04
Self-care	0.30	<.001
Engagement in usual activities	0.21	.011

Abbreviations: AL, allostatic load; BMI, body mass index; EQ-5D-5L, EuroQol 5-Dimension 5-Level.

**Table 3.** Multivariable Analysis of Sample Characteristics and AL Score

Characteristic	Correlation coefficient, *r* (95% CI)	*P* value
Educational level		
Less than high school	0.48 (−0.17 to 0.42)	.15
High school	0.34 (−0.11 to 0.80)	.14
Beyond high school	1 [Reference]	NA
Sex		
Men	0.63 (0.19 to 1.08)	.005
Women	1 [Reference]	NA
Patient Health Questionnaire–9	−0.01 (−0.07 to 0.05)	.89
Impact of Events Scale	0.015 (−.004 to 0.034)	.13
Life Events Scale	−0.01 (−0.24 to 0.22)	.94
EQ-5D-5L		
Mobility	0.37 (0.13 to 0.60)	.002
Self-care	−0.6 (−0.23 to 0.22)	.77
Engagement in usual activities	0.13 (−0.07 to 0.35)	.20

Abbreviations: AL, allostatic load; EQ-5D-5L, EuroQol 5-Dimension 5-Level; NA, not applicable.

### Overall Mortality

Variables associated with worse overall mortality on univariable analysis (Table 4) included higher AL (hazard ratio [HR], 1.63 [95% CI, 1.37-1.94]; *P* < .001), worse self-care (HR, 1.85 [95% CI, 1.37-2.49]; *P* < .001), and impaired mobility (HR, 1.40 [95% CI, 1.16-1.68]; *P* < .001). Conversely, higher educational attainment (HR for beyond high school, 0.45 [95% CI, 0.25-0.88]; *P* = .02) and treatment with chemotherapy plus immunotherapy (HR, 0.51 [95% CI, 0.27-0.98]; *P* = .04) or targeted therapy (HR, 0.40 [95% CI, 0.20-0.83]; *P* = .01) were associated with a lower overall mortality compared with lower educational levels and chemotherapy only, respectively. There was no association between Charlson Comorbidity Index and overall mortality on univariable analysis. On adjusted analysis, AL (HR, 1.43 [95% CI, 1.16-1.79]; *P* = .001) and low educational attainment (HR for less than high school, 2.11 [95% CI, 1.03-4.34]; *P* = .04 [reference value was beyond high school]) were associated with worse overall mortality (Table 5).

**Table 4.** Univariable Analysis Between Study Variables and Mortality

Variable	HR (95% CI)	*P* value
Age	1.00 (1.00-1.02)	.64
Sex		
Women	1 [Reference]	NA
Men	1.26 (0.80-2.00)	.32
Race		
Asian	1.73 × 10^−15^^a^	>.99
Black	1.37 (0.59-3.20)	.46
White	1 [Reference]	NA
Multiple races	1.37 (0.68-2.77)	.37
Other^b^	1.72 × 10^−15^^a^	>.99
Hispanic ethnicity		
No	1 [Reference]	NA
Yes	0.33 (0.05-2.43)	.28
BMI	1.00 (0.95-1.03)	.55
Income		
<$15 000	1 [Reference]	NA
$15 001-$25 000	1.89 (0.64-5.56)	.25
$25 001-$35 000	1.26 (0.47-3.33)	.65
$35 001-$50 000	2.11 (0.81-5.50)	.13
$50.001-$75 000	1.71 (0.61-4.80)	.31
$75 001-$100 000	2.11 (0.74-6.04)	.17
$100 001-$150 000	0.43 (0.11-1.74)	.24
$150 001-$200 000	1.16 × 10^−20^^a^	<.001
$200 001-$250 000	1.91 (0.38-9.59)	.43
≥$250 001	0.41 (0.05-3.51)	.42
Educational level		
Less than high school	0.57 (0.30-1.08)	.08
High school	0.47 (0.24-0.88)	.02
Beyond high school	1 [Reference]	NA
Marital status		
Not married	1 [Reference]	NA
Married	1.00 (0.64-1.57)	>.99
Charlson Comorbidity Index	0.94 (0.85-1.04)	.22
Smoker status		
Never	1 [Reference]	NA
Prior	1.45 (0.65-3.21)	.36
Current	1.86 (−0.76 to 4.55)	.72
Alcohol use		
No	1 [Reference]	NA
Yes	0.91 (0.59-1.43)	.71
Treatment		
Chemotherapy	1 [Reference]	NA
Chemotherapy and immunotherapy	0.51 (0.27-0.98)	.04
Immunotherapy	0.55 (0.27-1.12)	.10
Targeted	0.40 (0.20-0.83)	.01
Patient Health Questionnaire–9	1.02 (0.98-1.06)	.25
Generalized Anxiety Disorder 7	1.00 (0.95-1.04)	.86
Impacts of Events Scale	1.00 (0.99-1.01)	.58
Life Events Scale	1.21 (0.98-1.51)	.08
EQ-5D-5L		
Mobility	1.40 (1.16-1.68)	<.001
Self-care	1.85 (1.37-2.49)	<.001
Engagement of usual activities	1.14 (0.95-1.36)	.15
Allostatic load	1.63 (1.37-1.95)	<.001

Abbreviations: BMI, body mass index; EQ-5D-5L, EuroQol 5-Dimension 5-Level; HR, hazard ratio; NA, not applicable.

^a^ Because the value is small, the 95% CI is 0.

^b^ Consists of individuals who did not identify as Asian, Black, White, or multiple races.

**Table 5.** Cox Proportional Hazards Model to Predict Mortality

Variable	HR (95%CI)	*P* value
Allostatic load	1.43 (1.16-1.79)	.001
EQ-5D-5L		
Self-care	1.36 (0.92-2.01)	.12
Life events	1.22 (0.96-1.54)	.105
Educational level		
Less than high school	2.11 (1.03-4.34)	.04
High school	1.18 (0.66-2.15)	.57
Beyond high school	1 [Reference]	NA
Treatment		
Chemotherapy and immunotherapy	0.79 (0.38-1.62)	.52
Immunotherapy	0.94 (0.44-2.01)	.88
Targeted	0.84 (0.37-1.91)	.67
Chemotherapy	1 [Reference]	NA

Abbreviations: EQ-5D-5L, EuroQol 5-Dimension 5-Level; HR, hazard ratio; NA, not applicable.

### Sensitivity Analysis

The overall results of the sensitivity analysis were largely similar to those of the primary analysis. The correlation between AL and study variables were comparable with AL as quartiles or cutoffs (eTable 2 in the Supplement). Similarly, the association between study variables and mortality were consistent across both methods of AL calculations (eTable 3 in the Supplement). The direction of the association between AL and mortality was consistent with both AL calculation approaches.

## Discussion

To our knowledge, this cross-sectional study is the first to examine AL in patients with metastatic lung cancer. Our findings show that elevated AL was associated with adverse SDHs and higher overall mortality. Specifically, a 1-point increase in AL was associated with a 43% increase in the risk of death.

This analysis of the BLCIO cohort suggests there is evidence for AL to be conceptualized as a biological correlate of SDHs. Specifically, correlations between elevated AL and adverse SDHs (ie, low income, poor educational achievement, depressive symptoms, stressful life events, and poor functional status) provide some evidence of this conceptualization. Further, these results confirm existing studies in populations without cancer showing an association between adverse SDHs (ie, low income and poor educational attainment)^43,44,45^ and high AL. Nonetheless, additional research is needed to better delineate the mechanistic pathways through which SDHs affect AL and how that association influences oncologic outcomes (eg, mortality). Studies among patients without cancer suggest the association between AL and SDHs may be mediated through educational, material-financial, and behavioral pathways.^46^ For patients with cancer, additional studies in large diverse populations with a robust collection of SDH and AL biomarkers are needed to better delineate the role of SDH exposures as predictors, moderators, or mediators of AL.

The results of this study suggest multisystem physiologic dysregulation, operationalized as AL, may have implications for mortality in patients with advanced lung cancer. These study results are consistent with prior evaluations of AL in populations with^47^ and without^46^ cancer. In the review by Akinyemiju et al^20^ of the Reasons for Geographic and Racial Difference in Stroke study, a higher AL was associated with worse all-cause and cancer-specific mortality among patients who developed cancer in the cohort, adjusting for socioeconomic and sociodemographic factors. Similarly, in a meta-analysis by Mathew et al^47^ of studies evaluating AL in patients with cancer, a 1-unit increase in AL was associated with a 9% increase in mortality. These studies, in conjunction with ours, document the reliability of AL as a prognostic marker for mortality among patients with cancer.

The association between AL and overall mortality is significant when contextualized within study findings showing a correlation between AL and the Charlson Comorbidity Index but no correlation between the Charlson Comorbidity Index and mortality. The Charlson Comorbidity Index is a weighted index of comorbidities to estimate mortality.^48^ The positive correlation between the Charlson Comorbidity Index and AL is unsurprising because AL consists of secondary outcomes of the hypothalamic-pituitary-adrenal axis (eg, blood pressure) that are risk factors for diseases (eg, cerebrovascular accident) measured by the Charlson Comorbidity Index. The lack of an association between the Charlson Comorbidity Index and mortality suggests AL may contribute to clinical outcomes in ways that are different than existing measures in clinical practice. Further, it suggests AL might be measuring patterns of physiological response and possibly subsequent health risk beyond measures currently used in clinical practice.^22^

The results from this study highlight the need to use a diverse array of measures to understand the effect of SDHs among patients with cancer. Currently, most studies use patient-reported or neighborhood-level socioeconomic and sociodemographic characteristics to examine the association between SDHs and oncologic outcomes. This approach is limited, because SDH measurements will reflect only current living or working conditions and may not be representative of cumulative physiologic dysregulation secondary to life course exposure to adverse SDHs. Our study, in conjunction with the limited studies on AL in cancer, suggest life course SDHs may have implications beyond a particular time stamp or time frame. Consequently, biological correlates representative of life course exposures are needed to better understand the true effects of SDHs in patients with cancer and to enable the development of interventions to address these effects.

### Strengths and Limitations

The strength of this study is the evaluation of AL within an observational cohort study setting, where quality of care is similar across participants with complete, lengthy follow-ups. The SDH indicators across sociodemographic, psychological, and functional status domains strengthened the internal validity of the analysis along with the sample AL values approximating a normal distribution.

The study has some limitations. Some patients were excluded (n = 43) owing to not having all 9 biomarkers for the AL composite. Regardless, the AL distribution was normal, and the resultant number of participants was sufficient to detect a mortality effect. In addition, the biomarkers used were those found in prior studies.^26,27,28^ Despite literature establishing the construct validity of AL,^14,15^ investigators disagree on which physiologic systems need to be included, and no standard biomarkers are available for AL.^21,47^ Last, the study participants received care at a comprehensive cancer center and were predominantly White, limiting the generalizability of our findings.

## Conclusions

The findings of this cross-sectional study of patients with advanced NSCLC suggest that AL at study entry was associated with adverse SDHs and significantly higher mortality. These findings further suggest that AL could serve as a biological correlate of SDHs and a prognostic marker for mortality in patients with metastatic lung cancer. These results provide the framework for additional studies in other cancer sites examining AL as a biological correlate for SDHs and a future prognostic marker.

## Notes

Supplement. **eTable 1.** Means, SDs, and Ranges on Psychological and Functional Status Measures (N = 143) **eTable 2.** Correlation Between AL and Descriptive Characteristics, Psychological Variables, and Health Measures by Clinical Cutoffs vs Quartiles (N = 143) **eTable 3.** Univariable Analysis Between Study Variables and Mortality Using AL as Clinical Cutoffs vs Quartiles (N = 143) **eFigure.** Distribution of Allostatic Load Scores in Patients With Stage IV NSCLC (N = 143)
