# Error in Byline and Author Affiliations

**DOI:** 10.1001/jamanetworkopen.2023.6766

**Published:** 2023-03-17

In the Original Investigation titled “Association of Allostatic Load With Overall Mortality Among Patients With Metastatic Non–Small Cell Lung Cancer,”^1^ published July 7, 2022, Dr Reisinger’s surname was misspelled in the byline and author affiliations. This article has been corrected.
