# Supplementary material for: Association of Allostatic Load With Overall Mortality Among Patients With Metastatic Non–Small Cell Lung Cancer
Source: JAMA Netw Open. 2022 Jul 7;5(7):e2221626. doi: 10.1001/jamanetworkopen.2022.21626 (PMC9264034; doi:10.1001/jamanetworkopen.2022.21626)
Supplement: Supplement. — eTable 1. Means, SDs, and Ranges on Psychological and Functional Status Measures (N = 143) eTable 2. Correlation Between AL and Descriptive Characteristics, Psychological Variables, and Health Measures by Clinical Cutoffs vs Quartiles (N = 143) eTable 3. Univariable Analysis Between Study Variables and Mortality Using AL as Clinical Cutoffs vs Quartiles (N = 143) eFigure. Distribution of Allostatic Load Scores in Patients With Stage IV NSCLC (N = 143) [file jamanetwopen-e2221626-s001.pdf]

Supplementary Online Content

Obeng-Gyasi S, Li Y, Carson WE, et al. Association of allostatic load with overall mortality among patients with metastatic non–small cell lung cancer. *JAMA Netw Open*. 2022;5(7):e2221626. doi:10.1001/jamanetworkopen.2022.21626

eTable 1. Means, SDs, and Ranges on Psychological and Functional Status Measures (N = 143)
eTable 2. Correlation Between AL and Descriptive Characteristics, Psychological Variables, and Health Measures by Clinical Cutoffs vs Quartiles (N = 143)
eTable 3. Univariable Analysis Between Study Variables and Mortality Using AL as Clinical Cutoffs vs Quartiles (N = 143)
eFigure. Distribution of Allostatic Load Scores in Patients With Stage IV NSCLC (N = 143)

This supplementary material has been provided by the authors to give readers additional information about their work.

© 2022 Obeng-Gyasi S et al. *JAMA Network Open*.

eTable 1. Means, SDs, and Ranges on Psychological and Functional Status Measures (N = 143)

Measure	Mean (SD)	Median (interquartile range)
Patient Health Questionnaire-9	6.64±5.45	5(3-9)
Generalized Anxiety Disorder-7	5.52±5.58	4(1-7)
Impacts of Events Scale-Revised	16.9±15.69	12(6-22)
Life Events Scale	0.91±0.97	1(0-1)
Functional Status EQ-5D-5L		
Mobility	1.91±1.09	2(1-3)
Self-Care	1.31±0.64	1(1-1)
Engagement in Usual Activities	2.24±1.23	2(1-3)

eTable 2. Correlation Between AL and Descriptive Characteristics, Psychological Variables, and Health Measures by Clinical Cutoffs vs Quartiles (N = 143)

	AL Clinical Cut-Offs		AL Quartiles	
	Correlation Coefficient	p-value	Correlation coefficient	p-value
Age	-0.038	0.65	0.01	0.89
BMI	0.046	0.58	-0.07	0.39
Race	0.229	0.11	0.06	0.48
Hispanic	0.011	0.89	0.05	0.58
Income	-0.200	0.02	-0.141	0.11
Education	-0.228	0.006	-0.24	0.005
Marital status	0.042	0.62	0.03	0.71
Sex	0.193	0.02	0.13	0.14
Charlson Co-morbidity Index	0.062	0.46	0.23	0.0008
Smoker Status	01679	0.05	0.141	0.09
Alcohol Use	-0.099	0.24	0.02	0.84
Treatment	-0.333	0.002	0.23	0.010
Patient Health Questionnaire-9	0.235	0.005	0.25	0.002
Generalized Anxiety Disorder-7	-0.035	0.68	0.21	0.02
Impacts of Events Scale	0.247	0.003	0.27	0.001
Life Events Scale	0.304	<0.001	-0.0499	0.55
EQ-5D-5L				
Mobility	0.187	0.04	0.28	<0.001
Self-Care	0.304	<0.001	0.21	0.10
Engagement in Usual Activities	0.213	0.01	0.16	0.054

© 2022 Obeng-Gyasi S et al. *JAMA Network Open*.

eTable 3. Univariable Analysis Between Study Variables and Mortality Using AL as Clinical Cutoffs vs Quartiles (N = 143)

	AL Clinical Cutoff		AL Quartiles	
	HR (95% CI)	*p*-value	HR (95% CI)	*p*-value
Age	1.00 (1.00-1.02)	0.64	1.0 (0.99-1.02)	0.64
BMI	1.00 (0.95-1.03)	0.55	0.99 (0.95-1.02)	0.55
**Race**				
White	REF			
Black	1.37 (0.59-3.20)	0.46	1.37 (0.58-3.20)	0.463
Asian	1.73e-15	1.00	1.73e-15	1
Other	1.72e-15	1.00	1.72e-15	1
Multiracial	1.37 (0.68-2.77)	0.37	1.38	0.37
**Hispanic**				
No	REF			
Yes	0.33 (0.05-2.43)	0.28	0.34 (0.05-2.43)	0.281
Income				
1	Ref		Ref	
2	1.89 (0.64-5.56)	0.24	1.89 (0.64-5.55)	0.25
3	1.26 (0.47-3.33)	0.65	1.26 (0.47-3.33)	0.65
4	2.11 (0.81-5.50)	0.13	2.11 (0.81-5.50)	0.13
5	1.71 (0.60-4.79)	0.31	1.71 (0.61-4.80)	0.31
6	2.11 (0.73-6.6.04)	0.17	2.11 (0.73-6.04)	0.17
7	0.43 (0.11-1.73)	0.24	0.43 (0.11-1.74)	0.24
8	1.16e-20		1.16e-20	
9	1.91 (0.38-9.58)	0.43	1.91 (0.38-9.58)	0.43
10	0.41 (0.05-3.50)	0.42	0.41 (0.048-3.51)	0.42
**Education**				
<High school	0.57 (0.30-1.08)	0.08	Ref	
High School	0.47 (0.24-0.88)	0.02	0.79 (0.49-1.28)	0.34
>High School	REF		0.41(0.21-0.82)	0.01
**Marital status**				
Not married	REF			
Married	1.00 (0.64-1.57)	1.0	1.00 (0.64-1.57)	1.0
**Gender**				
Female	REF			
Male	1.26 (0.80-2.00)	0.32	1.27 (0.80-2.00)	0.32
Charlson Comorbidity Index	0.94 (0.85-1.04)	0.22	0.94 (0.85-1.04)	0.22
**Smoker Status**				
Never	REF			
Prior Smoker	1.45 (0.65-3.21)	0.36	1.45 (0.65-3.21)	0.36
Current Smoker	1.86 (-.76-4.55)	0.72	1.86 (0.76-4.55)	0.17
**Alcohol Use**				
No	Ref			
Yes	0.91 (0.59-1.43)	0.71	0.92 (0.59-1.43)	0.71
**Treatment**				
Chemotherapy	REF			
Chemotherapy and	0.51 (0.27-0.98)	0.04	0.51 (0.26-0.98)	0.04
Immunotherapy	0.55 (027-1.12)	0.10	0.54 (0.27-1.12)	0.10

© 2022 Obeng-Gyasi S et al. *JAMA Network Open*.

Immunotherapy Targeted	0.40 (0.20-0.83)	0.014	0.40 (0.20-0.83)	0.10
Patient Health Questionnaire-9	1.02 (0.98-1.06)	0.25	1.02 (0.98-1.06)	0.25
Generalized Anxiety Disorder-7	1.00 (0.95-1.04)	0.86	1.0 (0.95-1.04)	0.86
Impacts of events scale	1.00 (0.99-1.01)	0.58	1.00 (0.98-1.01)	0.22
Life events scale	1.21 (0.98-1.51)	0.08	1.21 (0.98-1.51)	0.08
**EQ-5D-5L**				
Mobility	1.40(1.16-1.68)	0.000	1.39 (1.16-1.68)	0.000
Self-Care	1.85(1.37-2.49)	0.000	1.85 (1.37-2.49)	0.000
Engagement of Usual Activities	1.14 (0.95-1.36)	0.15	1.13 (0.95-1.36)	0.15
**Allostatic load**	1.63 (1.37-1.95)	0.000	1.19 (1.01-1.38)	0.03

eFigure. Distribution of Allostatic Load Scores in Patients With Stage IV NSCLC (N = 143)

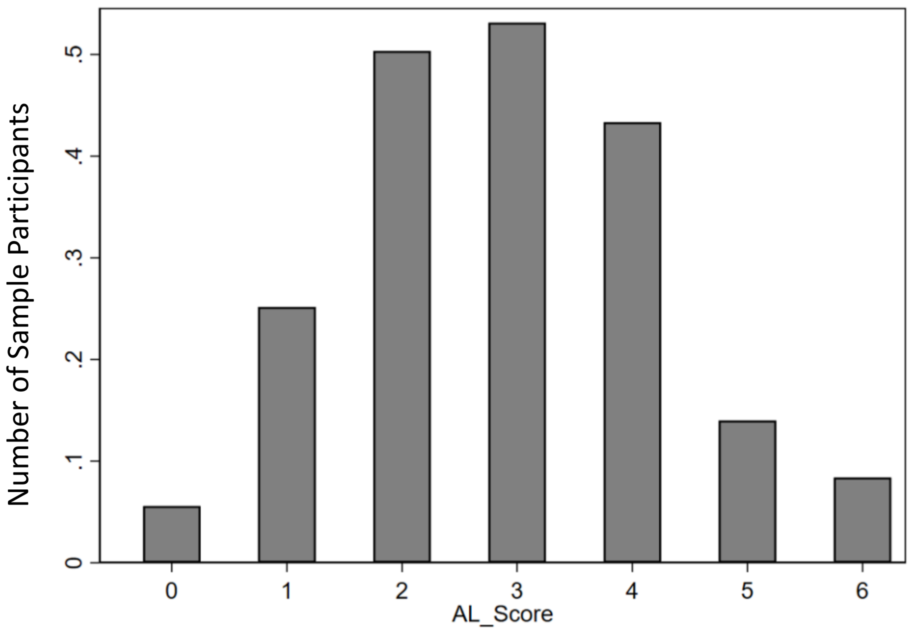

Variable	Observations	Mean	SD	Min	p25	p50	p75	Max
**AL Score**	143	2.90	1.37	0	2	3	4	6

© 2022 Obeng-Gyasi S et al. *JAMA Network Open*.
